# Experimental Studies of Fluidized Bed Calcination of Granulated Clay Material

**DOI:** 10.3390/ma17102185

**Published:** 2024-05-07

**Authors:** Katarzyna Kaczyńska, Piotr Pełka

**Affiliations:** Czestochowa University of Technology, Faculty of Mechanical Engineering and Computer Science, Institute of Thermal Machinery Al. Armii Krajowej 21, 42-201 Czestochowa, Poland; kmc.wimii@pcz.pl

**Keywords:** clay raw materials, fluidization, calcination, thermal enrichment

## Abstract

The work presents a detailed analysis of the possibilities of the thermal processing of clay raw material granulates in a fluidized bed reactor powered by coal fuel. Potential customers of calcined granulates include the following: plants producing refractory materials for the steel industry, producers of refractory concrete, sanitaryware plants, tile plants, large-size tile plants, industry abrasives, chemicals, paints, paper, food and medical industries and others. The advantage of the presented fluid bed calcination technology is the possibility of the continuous operation of the reactor and the short time of the material in the bed, compared to the previously used methods of calcination in a shaft and rotary kiln, which lasts less than twenty minutes in the temperature range of 650–850 °C. During the experimental studies of calcination in the fluidized bed layer, the influence of the type of coal, its particle size and the mass share of coal in the feed mixture on the calcination process and the final product obtained was analysed. As a result of the conducted research, it was proven that solid fuels such as anthracite and steam coal type 31.2 (flaming) can be successfully used in the fluidized bed calcination process of clay materials. The key parameter determining the fluidized bed calcination process is the fuel particle distribution.

## 1. Introduction

Clay minerals are mostly represented by layered silicates and aluminosilicates, and depending on the mutual positions of the layers, they may belong to the following silicates: two-layer silicates with a 1:1 structure type (e.g., kaolinite) and three-layer silicates with a 2:1 structure type (e.g., illite) [1,2]. The resources of clay rocks are abundant and extensive. They are common in various parts of the Earth’s crust subject to weathering processes (mainly chemical) in temperate climate conditions [3]. Clay minerals have been used since prehistoric times in ceramics, pottery and sculptures. Currently, the use of clay raw materials in industry is estimated at hundreds of millions of tons because they are minerals with very versatile properties. The reason for this is also their great diversity and the simplicity of their modification. In recent years, there has been a significant increase in interest in materials obtained from thermal processing of clay raw materials.

Calcination is a heat treatment process in which the processed material is heated to its decomposition temperature which releases water and other volatile components, e.g., CO_2_ from carbonates contained in the raw material. When raw clays are exposed to increasing temperatures, three main phenomena can be distinguished related to the aluminosilicate material: dehydration, dehydroxylation and recrystallization. Dehydration is the release of molecular water that may be adsorbed or trapped in a pore or channel structure or that is associated with interlayer cations common in most clay minerals of a 2:1 structure type [4]. The size and range of dehydration temperatures depend on many factors such as storage conditions, the crystallinity of clay minerals and the nature of cations in the interlayer area [5]. At temperatures of 400–1000 °C, most clay minerals undergo dihydroxylation or, in other words, release water bound in the structure in the form of OH groups. The dehydroxylation temperature depends on the structure of the mineral. Thus, for a 1:1 structure type clay mineral, a disordered, metastable material can be obtained, while 2:1 structure type clay minerals retain some of the initial crystal structure after dehydroxylation and require higher calcination temperatures to activate calcination. At temperatures higher than 850 °C, recrystallization occurs, which indicates the conversion of structurally disordered, potentially reactive phases into more stable, high-temperature phases. A crystalline aluminosilicate spinel is formed, which transforms into mullite with a further increase in temperature.

Clay materials subjected to the calcination process may exhibit pozzolanic properties [6,7,8,9], and their use in construction is increasing because they are available in adequate quantities and can improve both the physical and chemical properties and durability of concrete [10,11,12,13]. Calcined kaolinite—metakaolinite—is commonly used as a supplementary cementitious material (SCM) and in cement production [14,15,16]. Recent achievements, including the use of calcined clays in a cement system in combination with limestone [17,18,19], their use as precursors for alkali-activated cement [20] and/or geopolymers [21], their use in the production of lime–pozzolanic cement [22] and combining them with magnesia for the production of hardened cement [23], indicate that the potential of using calcined clay minerals as building materials is huge.

### Methods of Calcination of Clay Raw Materials

The calcination of clay materials can be accomplished using a number of techniques. In the past, shaft kilns were commonly used [24], whereas now, calcination in a rotary kiln is more often used. The calcination temperature using these technologies is approximately 1000 °C. They are characterized by a long calcination process time (up to several hours), which does not guarantee the complete combustion of the carbon accompanying clay raw materials. Due to difficulties in temperature control, recrystallization and the formation of a new stable phase may occur (mullite, cristobalite, etc.), leading to a decrease in the reactivity of the calcined product. A novelty is the flash calcination method (flash heating), which involves calcining particles of very finely ground clay raw material in seconds (0.5–12 s). However, this method requires drying and grinding the original clay material into a form where 90% of the particles will be smaller than 2 µm [6,25]. Innovative technologies for the calcination of clay raw materials include solar calciners [26,27], microwave calcination [28,29] and plasma calcination [30], but these technologies have many limitations in terms of implementation on an industrial scale.

An alternative method of calcining clay raw materials is calcination in a fluidized bed. So far, fluidized bed furnaces on an industrial scale have only been used for burning limestone in the production of quicklime [24,31]. However, research carried out on a laboratory scale [31,32,33,34,35,36] and the pilot installation [37] demonstrated the potential of this technology to be used in the calcination of clay raw materials. Fluidized bed technology offers potential benefits in terms of energy (fuel) savings, a high heat transfer coefficient, the intensive contact of hot gases with clay material particles, the guarantee of a uniform temperature and product homogeneity, a short process time and the easy operation and maintenance of the installation.

A characteristic feature of the innovative fluidized bed calcination technology examined in this work is the possibility of the continuous operation of the reactor. This means the continuous supply and collection of the product, thus allowing the processing of a large amount of raw material in a short time without interrupting the process. This fact indicates the possibility of using this technology in the calcination of clay granulates on an industrial scale. Another very important feature of the proposed technology is the so-called fuel flexibility. The choice of energy source is one of the most important factors influencing the energy efficiency of the production process, as well as the properties of the final product. This work analyses the possibilities of the thermal processing of clay granulates in a fluidized bed reactor using coal fuel. This choice was motivated by the fact that the clay raw material industry is often linked to the coal mining industry. Another motivation was that of the findings made in previous studies [36], based on which it was stated that during fluidized bed calcination, carbon–organic pollutants are completely burnt. An additional advantage of these fuels is the fact that coal combustion processes in a fluidized bed have been widely studied in terms of the use of this technology in power boilers (fluidized bed boilers).

In the calcination process of clay raw materials, the physicochemical properties of the fuel are very important. The choice of fuel determines the quality of the final product. In the paint and paper industry, the use of diesel and fuel oil is preferred because the most important process requirement is the whiteness of the calcined product. The use of other fuels may cause colour changes or coloured inclusions in the final product. The colour of the calcined product is not that important in the construction industry where calcined clay materials are used only to improve the physical, chemical properties and durability of concrete [10,11,12,13]. For this reason, coal or petroleum coke may be used in the calcination of clay raw materials for the construction industry. There are also known cases of the use of biomass and other waste fuels [38]. Therefore, in the process of the calcination of clay raw materials, the composition of the fuel, especially the presence of impurities in it, is extremely important. Contaminants such as sulphates, chlorides, etc., may participate in hydration with cement and affect the durability of concrete.

In this work [39], the phenomena of the release and ignition of volatile parts, as well as the ignition and combustion of the char of lignite, hard coal and anthracite particles with diameters of 4–9 mm in a two-dimensional fluidized bed, were investigated. It has been shown that carbon particles tend to float on the surface of the bed during the release and combustion of volatiles. At 21% oxygen concentration, the bed temperature required to ignite the volatiles was approximately 680 °C. It was found that the ignition temperature of the char of lignite particles was 220 °C, while for anthracite particles, the bed temperature should be at least 600 °C. The average heating rates of coal particles were from 12 °C/s for particles of 9 mm and 120 °C/s for particles d = 4 mm at a bed temperature of 650 °C and from 20 °C/s (d = 9 mm) to 200° C/s (d = 4 mm) at 850 °C.

Solid fuel containing volatile components (e.g., hard coal) is characterized by two processes and two auto-ignition temperatures: auto-ignition and the auto-ignition temperature of volatile components and auto-ignition and the auto-ignition temperature of the char. The heating of the combustible mixture from the initial temperature of the particle to its self-ignition temperature takes place within a specific time. This time is called the induction time, delay time or ignition delay time [40]. As stated in [39], the delay times for the ignition of volatiles (1–20 s) and the ignition of the char (5–200 s) decrease at higher bed temperatures. The typical values of the flame extinction time (volatile burning times) are 15 secs for a coal particle with a diameter of d = 4.7 mm and 40 s for a coal particle with a diameter of d = 8.6 mm.

## 2. Research Methodology

In the first stage of the experimental research described in this work, the parameters of the tested clay material and the parameters of the fluidized layer formed from it were determined, which were important for calcination. Subsequently, calculations were made of the demand for the total heat of the fluidized bed calcination in the reactor. In the subsequent stage, a technical and elemental analysis of coals was performed, and the minimum fuel demand in the calcination process was calculated.

During basic experimental studies of the calcination of selected clays in a reactor fuelled with coal fuel, the influence of the type, particle size and mass share of coal in the feed mixture on the calcination process and the final calcination product was analysed. Two types of coal fuel were used as follows: hard coal type 31.2 (flaming) and anthracite. Anthracite was chosen due to its expected (average 2–12%) and low mineral content, usually 2–8%. The low ash content obtained after burning anthracite ensures the purity of the final calcination product. Hard coal type 31.2 was chosen as an example of typical steam coal. It is available on the market and commonly used during combustion in fluidized bed boilers.

The optimal temperature range for kaolinite calcination, given in [41], is 650–850 °C. Therefore, research assumed that the calcination temperature in the reactor chamber would be 850 °C. According to the literature data [38], it is a temperature sufficient to ignite selected coal fuels with the particle size determined in the tests.

In the final stage of the work, tests were carried out to verify the calcination process. The loss on ignition and the degree of calcination were determined, and changes in the particle distribution of the material as a result of fluidized bed calcination were analysed.

### 2.1. Determination of the Degree of Calcination

As mentioned in the introduction, the dehydroxylation temperature range covering the full calcination of typical clay raw materials, i.e., those rich in kaolinite and illite, is 100–850 °C [42].

The determination of the loss on ignition must be preceded by the determination of the dry weight of the sample because the loss on ignition refers to it [42]. Therefore, in order to determine the dry mass of the sample, granules weighing 1 g were initially deprived of moisture by drying at a temperature of 105 °C. Subsequently, a sample of dry granulate was calcined in a muffle furnace at 100–850 °C and roasted at 850 °C for 1 h. The loss on ignition was calculated by comparing the dry weight of the sample before roasting to its weight after roasting. The analysis was performed in triplicate, and the average result of the loss on ignition of the raw material in the calcination process was determined. In the same way, the ignition loss of all granulate and coal mixtures was determined each time after their calcination process.

The degree of calcination of the material after calcination (*C*) is determined by the following relationship:

(1) $$C=1-{LOI}_{AC}/{LOI}_{RM}*100\%\;\left[\%\right]$$

where

C—the degree of calcination of the material after calcination;
${LOI}_{AC}$—the loss on ignition after calcination;
${LOI}_{RM}$—the loss on ignition of the raw clay material.

### 2.2. Characteristics of the Research Material

**Figure 1:**
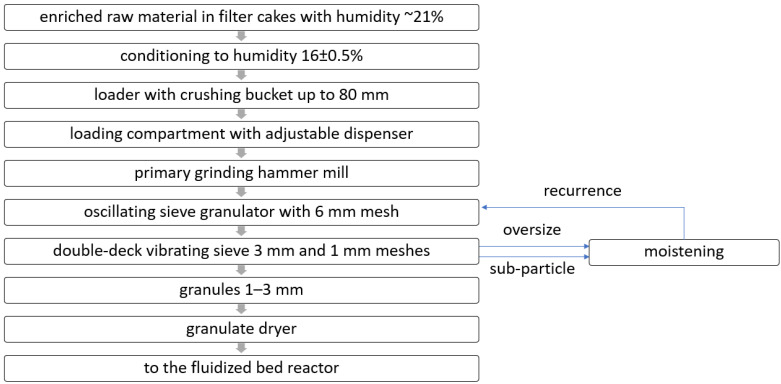
Block diagram of fluidization granulate production.

**Figure 2:**
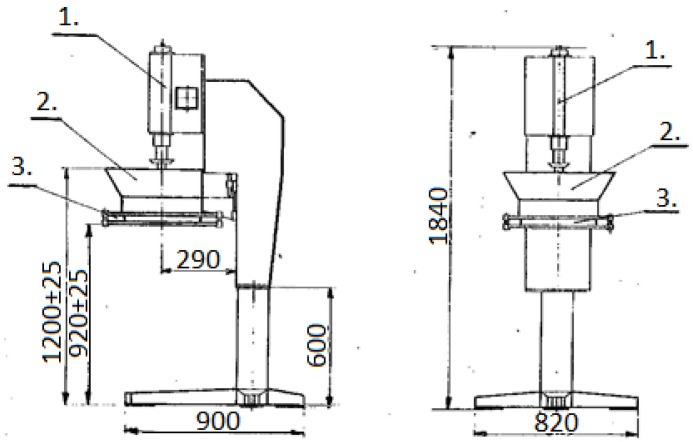
Reciprocating rotating sieve machine for granulation. 1—reciprocating and rotating mechanism, 2—power supply bowl, 3—attaching the sieve to the pulper.

Due to the fact that the primary clay material consists of sub-fine particles (less than a dozen or so µm), which are impossible to fluidize, an innovative solution was decided on in this study, which involved the prior preparation of clay granulates. The block diagram for the production of granules for testing is shown in Figure 1. In the granulation process, it is required that the enriched raw material has a standardized moisture content within a narrow range, i.e., 16 ± 0.5%. In order to ensure such humidity, the filter cakes of the enriched raw material obtained in the filtration process of the suspension of the enriched clay raw material were conditioned in atmospheric conditions and, after obtaining the required humidity, were pre-crushed in a semi-technical hammer mill with a diameter of 800 mm, which was equipped with a 16 mm escape partition. The pre-crushed raw material was then granulated on a sieve granulator, as shown in Figure 2. The reciprocating rotary granulator consisted of a base column on which the reciprocating rotary mechanism was mounted (1) and also a bowl for the pre-crushed raw material for granulation (2), vertically adjustable within ±25 mm in relation to the reciprocating rotary grinder with a ring and (3) the mounting of the grinding screen.

In tests to obtain granulates, woven sieves with a mesh size of 3, 4 and 6 mm and a perforated sieve with round holes of 6 mm diameter were used. The granulate obtained from the grinder was spread into particle classes on a vibrating sieve. The undersized and oversized particles obtained from the process of spreading granulates were moistened, mixed until homogenized and returned to grinding so that the entire raw material intended for granulation was finally obtained in the form of granules of the assumed particle class.

The sown granulates of the required particle class were dried on a screw dryer with a heated screw pipe, which was constructed especially for the purpose of observing the drying process under screw transport conditions. In these conditions, the granulate was surrounded, especially during the first passage through the dryer screw, when the granulate humidity was the highest. The drying of the granulate below 5% humidity was achieved after passing the granulate through the dryer 4 or 5 times.

Therefore, the research material consisted of granulated clays enriched from Polish lignite deposits, obtained in the process of purifying mud water, which was comprehensively enriched with high-alumina kaolin accompanying vein quartz deposits. In the enriched product, the mass ratio of substrates was 1:1. Enrichment aims to increase the Al_2_O_3_ content and reduce the Fe_2_O_3_ content. Table 1 presents the results of tests on the chemical composition of the material, which were performed for materials without transient moisture and crystal-bound water [43].

The technical analysis of the clay raw material was carried out in accordance with the Polish Standards applicable to solid fuels [44,45,46] The results of the technical analysis are presented in Table 2.

The ignition loss of the raw clay material was 13.92%.

The bulk density of the granules was determined according to the Polish Standard [47] and amounted to 1031.59 kg/m^3^.

**Figure 3:**
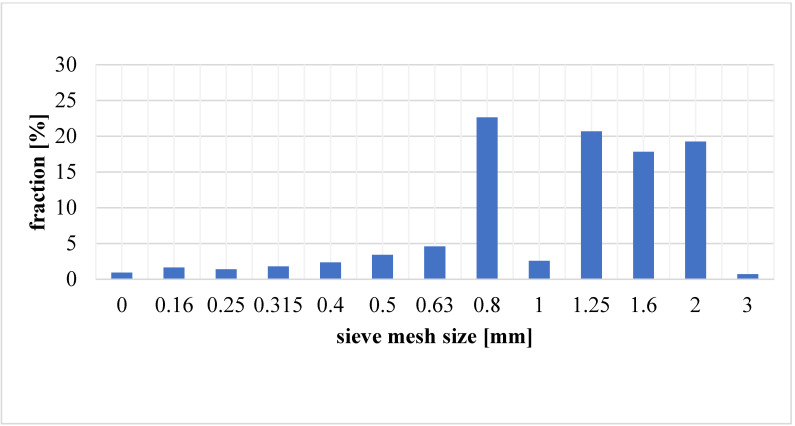
Residual clay mass on the sieves.

**Figure 4:**
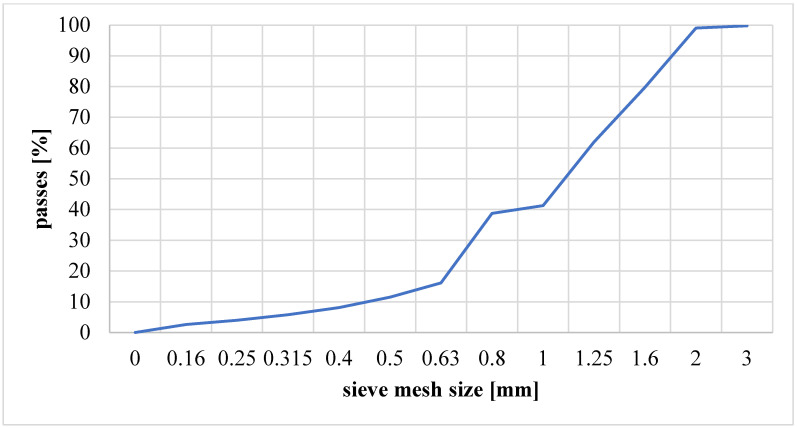
Cumulative graphs.

A sieve analysis was performed for the clay material, and average distributions showing the percentage of clay mass remaining on the sieves were determined (Figure 3), as well as cumulative graphs (Figure 4).

### 2.3. Fluidization Parameters of Clay Material

Based on the particle analysis of the clay material, as illustrated in Figure 4, the equivalent diameter of the so-called Sauter diameter (d_a_) was established, which was equal to 0.00125 [m]. This subsequently allowed the determination of the Archimedes number (Ar), the Reynolds number (Remf), the minimum fluidization velocity (Umf), the lifting velocity (U_t_) and the pneumatic transport velocity (U_tr_) [48]. After analysing the particle distribution and determining the mass density, the tested clay material was assigned to class “B” of bulk materials in the Geldart classification [49]. On this basis, it was assumed that the minimum fluidization velocity would be equal to the minimum velocity of the bubble layer (U_mb_). Table 3 presents the results of the calculations of fluidization parameters for ambient temperature and the specific calcination temperature of 850 °C.

### 2.4. Experimental Stand

**Figure 5:**
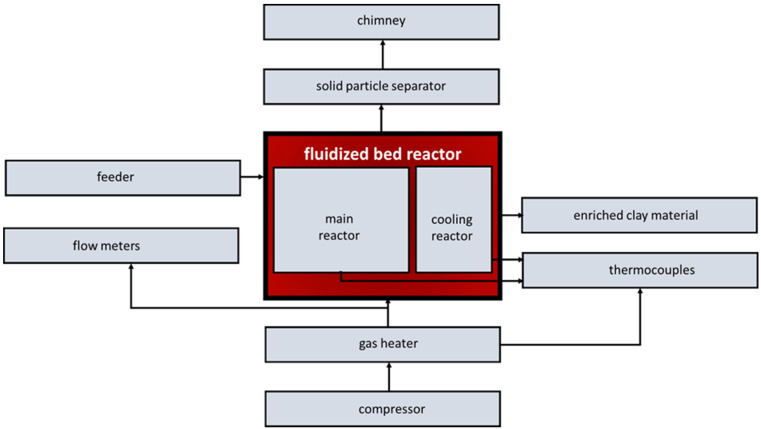
Block diagram of the station for fluidized bed calcining of clay material.

Experimental tests on calcination were carried out at the station described in [36]. For individual tests, the appropriate homogeneous mixtures of raw clay granules and selected ones with different particle size and mass fraction were prepared. The design of the laboratory station enabled fuel to be introduced simultaneously with the clay material through a feeder located in the upper zone of the main reactor, as shown in Figure 5. The total time of fluidized bed calcination, i.e., the time of the mixture in the main chamber of the reactor, was set at 16 min based on previous tests because for this time, a calcination degree of >95% was achieved for different clay granulates.

In order to obtain the proper calcination temperature and maintain a constant temperature between experiments, the reactor was powered by 4 electric heaters with a power of 1.5 kW each, mounted on the walls of the main chamber of the reactor and air-heated to 850 °C from the heater used to fluidize the bed. During the heating process, previously calcined granulate was fed into the reactor. After achieving the stable fluidization of the bed and the appropriate temperature in the main chamber of the reactor, the calcined material in the feeder tank was replaced with the appropriate mixture of fuel and raw granulate. When the mixture filled the main chamber of the reactor, the heaters in the main chamber were turned off and, in some justified cases, in the air heater too. After calcination in the main chamber of the reactor, the material was fed to a cooling chamber supplied with air at ambient temperature to cool it. The cooled material left the reactor by gravity.

In the first stage of basic research, two experiments were carried out on the calcination of pure raw granules with a moisture content of 4.32% and 0.6%, with the station being electrically powered. The second stage of the research was devoted to examining the calcination of a mixture of granules and anthracite hard coal with different particle sizes and different mass fractions—a total of 10 experiments. During this time, the electric heaters were turned off each time.

In order to determine the amount of fuel required for the calcination process, a technical and elemental analysis of the tested coals was performed. Calculations of the heat flow demand in the calcination process in the reactor at 850 °C were carried out, and on this basis, the minimum fuel demand in the tested process was calculated.

After completing the examination of the calcination process, tests were carried out each time to verify the calcination process, in which the loss on ignition and the degree of calcination were determined, and the particle distribution of raw and calcined granules was compared.

## 3. Experimental Research on Feeding a Fluidized Bed Reactor with Carbon Fuels

### 3.1. Fuel Characteristics

As mentioned earlier in the article, two types of coal fuel were used during the tests: hard coal type 31.2 (flaming) and anthracite. The analysis of both fuels was performed in accordance with Polish Standards applicable to solid fuels, and its results are presented in Table 4 and Table 5.

Due to the unusual composition of anthracite 1 (high ash content above 10%), it was decided to additionally use a second anthracite (anthracite 2) from another source with a lower ash content in the tests.

Comparing the obtained results of the technical analysis of anthracites with the literature data [39], where the typical composition of anthracites is 86–98% fixed carbon, 2–12% volatile substances and 3–6% moisture content and their calorific value is 34.89 KJ/kg, it can be seen that the anthracites selected for testing were characterized by a significantly lower content of a solid combustible part, which was within the range that is typical for hard coal. The results of the technical analysis of the selected energy of hard coal were very close to the values typical for hard coal of this type.

It should be emphasized that both anthracites were characterized by low sulphur and nitrogen content. Th elemental analysis showed a content of <1% for both elements.

The obtained results facilitated the calculation of the minimum fuel demand for the calcination process at a temperature of 850 °C, which, in the case of anthracite 1, is 10.4% of the mass fraction of the mixture, for hard coal type 31.2 (flaming), 11.5%, and, in the case of anthracite 2, 9.5%.

### 3.2. Heat Flow Demand for Calcination

In order to determine the total heat flow demand for the calcination process at 850 °C, the heat flow demand was calculated as follows:

- For the process of heating the total moisture of the clay material, evaporation, superheating of water vapour and to heat the clay material;
- To heat the air;
- For the calcination process.

**Figure 6:**
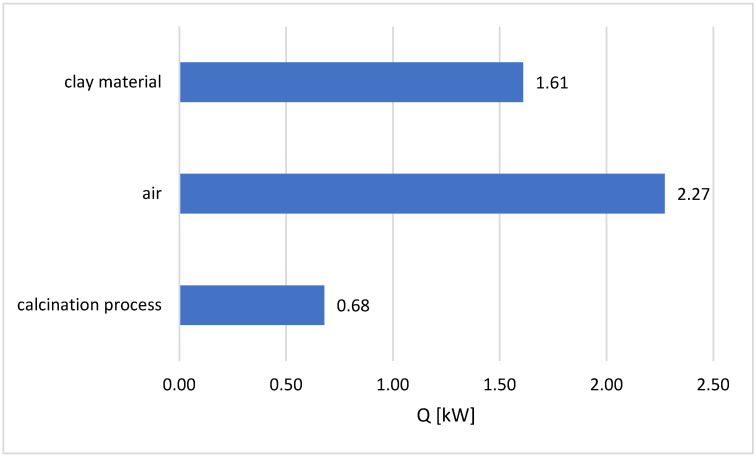
Heat flow demand in the calcination chamber—summary.

This was carried out assuming that the endothermic effect of calcination was 485 [kJ/kg], as illustrated in Figure 6. As a result of the calculations, it was determined that the total heat flow demand was 4.56 [kW] in the process.

### 3.3. Results of Experimental Studies

**Figure 7:**
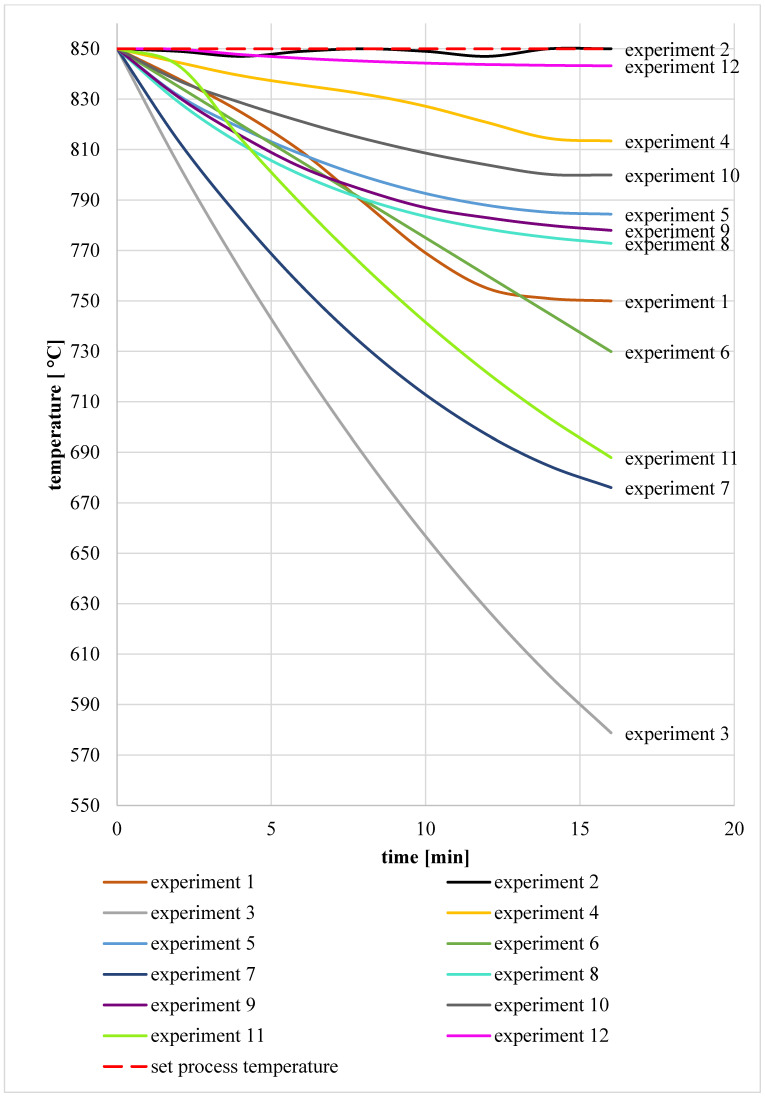
Temperature in the reactor chamber during clay material calcination experiments while feeding the station with coal fuels and electric heaters.

In order to control the calcination and fluidization process during the experiments, the temperature was measured in the main chamber of the reactor and the structure of the layer was observed during the process through sight glasses placed in the wall of the main chamber of the reactor. The recorded temperature courses in the reactor chamber during the calcination processes are shown in the chart, namely, Figure 7. After each experiment (both with electric power and with the use of fuel), the calcination loss and the degree of calcination were determined. The results of these determinations are presented in Table 6. Additionally, the table shows the actual fluidization velocity measured during the process and information about which power sources of the station were turned off during the experiment.

As mentioned in Section 2.4, in the first stage of experimental research, the granulate calcination process was carried out in a reactor powered by the electric heaters and air from the heater heated to 850 °C, which was used to fluidize the bed. Clay granulate with a moisture content of 4.32% was selected for the calcination tests. When measuring the temperature in the main chamber of the reactor (experiment no. 1), a significant drop was observed, reaching 106 °C. This decrease was observed from the beginning of the material administration of approximately 10 min. After this time, the temperature stabilized at 744 °C. The fluidization process was stable throughout the experiment. Despite the observed drop in the process temperature, the material after calcination was characterized by a high degree of calcination of 97.92%.

In order to identify the reason for the temperature drop during the calcination of raw clay granulate with the station being electrically powered, it was decided to calcinate the material without moisture. When experiment 2 was performed, no further temperature drop was observed. The process temperature oscillated around the assumed value. The material after calcination was characterized by a higher degree of calcination than in the previous study (no. 1), namely, 99.91%.

In the second stage of the experimental research, calcination tests were performed on raw granules with a moisture content of 4.32%, with two anthracites and hard coal with different particle sizes and different mass fractions. As mentioned in Section 2.4, the fuel was introduced simultaneously with the clay material in the form of a homogeneous mixture through a feeder located in the upper zone of the main reactor, as displayed in Figure 5. When the mixture filled the main chamber of the reactor, the heaters in the main chamber were turned off. In some cases, when turning off the heaters did not cause a temperature drop, it was also decided to turn off the fluidizing air heater.

**Figure 8:**
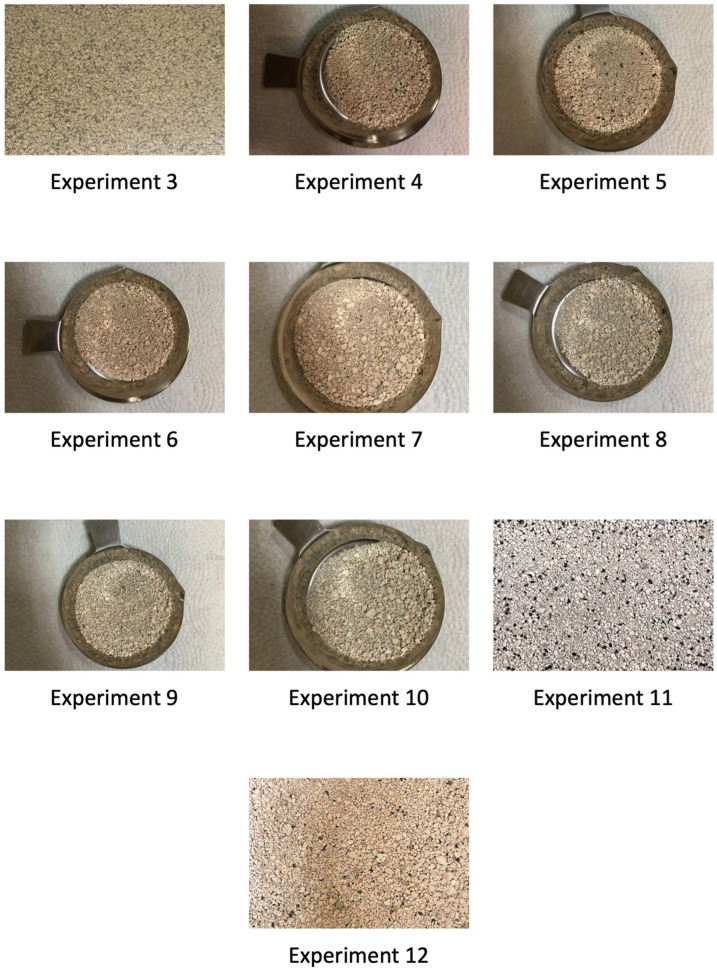
Materials after the calcination process while feeding the station with coal fuels.

The first tested mixture was a mixture of granules and anthracite 1 with a particle size of <3 mm (experiment no. 3). It was decided to try calcination using fuel without its prior grinding, with a particle size obtained from the supplier, i.e., <3 mm. Based on the calculations [39], it was determined that 16 min was sufficient for the complete combustion of anthracite particles of this diameter. The anthracite content resulting from the balance calculations was 10.5% of the mass share of the batch mixture. During this experiment, a continuous decrease in temperature was observed in the reactor chamber, which, at the end of the experiment, was only 578 °C. A high share of unburnt anthracite in the material after calcination was observed, as indicated in Figure 8. It was considered that the reason for the anthracite residue was the fuel feeding system used in the station, where the fuel mixed with the raw material was introduced directly into the bubble layer of the bed. Due to the small size of the site, this resulted in local cooling of the layer in this area. Through the sight glass in the reactor wall, an uneven ignition of fuel particles delivered to the station was observed. Due to the extension of the ignition time, it was difficult to completely burn the largest particles of anthracite 1. In this work [39], it was proven that the effect of the inert material of the bed at a temperature of 850 °C affects the delay in the combustion time of the volatile parts of coals and, among others, anthracite. Due to the temperature in the chamber at <650 °C and the very large amount of unburnt carbon, for obvious reasons, the determination of the loss on ignition of the material after calcination was not carried out. Due to problems with the complete combustion of coal during test no. 3, it was decided to reduce the size of fuel particles in subsequent tests (no. 4 and no. 5). At the same time, it is important to remember that due to the fluidization process, the particle distribution of the fuel should be adapted to the particle distribution of the clay granulate. Owing to this fact, the anthracite particle size range was reduced from 1 to 0.75–1.6 mm. Similarly to experiment no. 3, the content of anthracite 1 was 10.5% of the batch mixture. During test no. 4, a significantly smaller temperature drop was observed compared to test no. 3, and after 16 min, the temperature in the chamber settled at 810 °C. It should be noted that this temperature was higher than in the case of the calcination of raw granules while feeding the main chamber of the reactor with electric heaters (experiment no. 1). The calcination degree result was determined to be 97.73%. In the case of test no. 5, after the positive result of calcination in test no. 4, it was decided to additionally turn off the air heater. As a consequence, a slightly greater drop in temperature was observed in the main chamber than in the case of experiment no. 4. Nevertheless, after 16 min, the temperature in the chamber was 780 °C, i.e., the temperature was maintained higher than during the electric power supply (experiment 1). In the case of test no. 5, the degree of calcination was 95.74%. In order to optimize the process and increase the degree of calcination, in tests no. 6 and no. 7, it was decided to change the particle distribution again to the range of 0–0.75 mm. The anthracite content in the mixture remained unchanged. This time, a slightly larger one was observed than in studies no. 4 and no. 5 in terms of the temperature drop in the calcination chamber. After 16 min, the temperature was 730 °C. Since no unburnt fuel residues were noticed in the material after calcination, as indicated in Figure 8, it was concluded that excessive fuel fragmentation was responsible for the drop in temperature in the chamber. As a result, the smallest particle fractions along with post-reaction gases were blown out of the reactor chamber, which had not been burnt in the bed and caused a loss of incomplete combustion. Despite the observed drop in temperature in test no. 6, the calcination degree determined was 95.18%. During test no. 7, as in the case of test no. 5, both the heaters and the air heater were turned off. This resulted in an even greater reduction in the process temperature. After 16 min, the temperature in the main chamber was only 676 °C. Once again, no unburnt anthracite was observed in the material after calcination, as shown in Figure 8. However, a satisfactory degree of calcination was obtained, amounting to 93.76%.

The next three tests (no. 8, 9 and 10) were carried out using hard coal type 31.2. In the case of experiment no. 8, due to the lower ignition temperature of the particles of this type of coal [39], a different particle size range was established than in the case of anthracite. The particle size was 0.75–2 mm and the fuel content was 13% of the mixture. During the experiment, the temperature in the main chamber initially decreased, as in previous tests. The lowest temperature recorded was 772 °C. A small amount of unburnt carbon was observed in the material after calcination, which is indicated in Figure 8, and the degree of the calcination of the material was 96.41%. Searching for the reasons for the temperature drop in the first minutes of the experiment, it was decided to reduce the particle size, thus shortening the ignition induction time. The particle size of hard coal in subsequent experiments was in the range of 0.75–1.6 mm (as in the case of anthracite 1). Additionally, due to the fact that the calculations of the minimum fuel demand did not take into account the heat losses in the process through radiation, it was decided to increase the share of coal from 13 to 15% in test no. 9 and then from 15 to 17% in test no. 10. As a result, the lowest recorded process temperatures were 778 °C and 813 °C, respectively. The presence of unburnt fuel in the material after calcination was not observed, as illustrated in Figure 8. For both cases, a very good result of the material calcination degree was obtained at the level of >99%.

**Figure 9:**
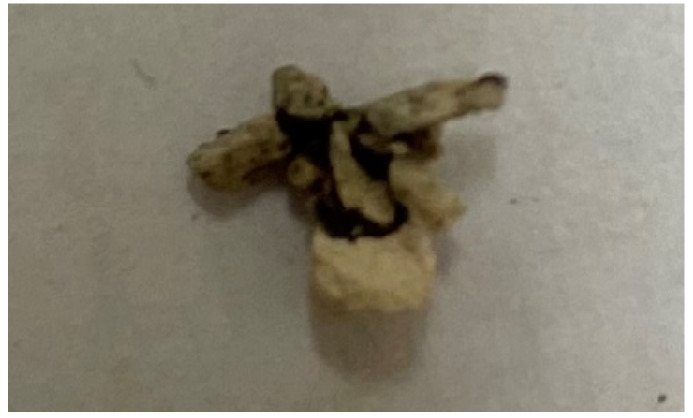
Example of sinter produced in the reactor, experiment 11 (diameter approximately 6 mm), which caused defluidization of the bed and problems with the feeder operation.

In the final stage of the experimental research, the station was fed with anthracite 2. In the case of test no. 11, the station was fed with anthracite 2 with a particle size of 1–1.6 mm. Due to the positive effect of increasing the fuel share on the calcination process, the carbon content was set at 17% in experiments no. 9 and no. 10. Despite the previous positive effects obtained by increasing the fuel share compared to the value calculated during experiments with hard coal (experiments 9 and 10), contrary to expectations, this time, the temperature in the main chamber decreased. Shortly after starting the experiment, there were problems with maintaining a stable fluidization process and problems with the feeder operation. After a while, the defluidization of the deposit was observed. A significant amount of unburnt coal particles, agglomerates and sinters were observed in the material after calcination, as shown in Figure 8 and Figure 9. Based on the analysis, it was found that the agglomerates were produced as a result of the release of tar substances on the anthracite surface at a temperature below the fuel ignition temperature [50]. The determination of the loss on ignition in the case of test no. 11 confirmed that the calcination process took place to a small extent only in the initial phase of the experiment. The degree of calcination was only 36.34%. In the case of test no. 12, due to the failure of experiment 11, the size of coal particles was reduced and the share of coal in the mixture was reduced to limit the formation of a large amount of agglomerates, which led to the defluidization of the bed. The input material was a mixture of clay material and anthracite with a particle size of <1 mm, where the carbon content was 15% of the mixture. In this case, the temperature in the main chamber remained at a level close to the set process temperature. A small amount of unburnt carbon particles was observed in the material after calcination, as indicated in Figure 8. The degree of calcination was 93.76%.

**Figure 10:**
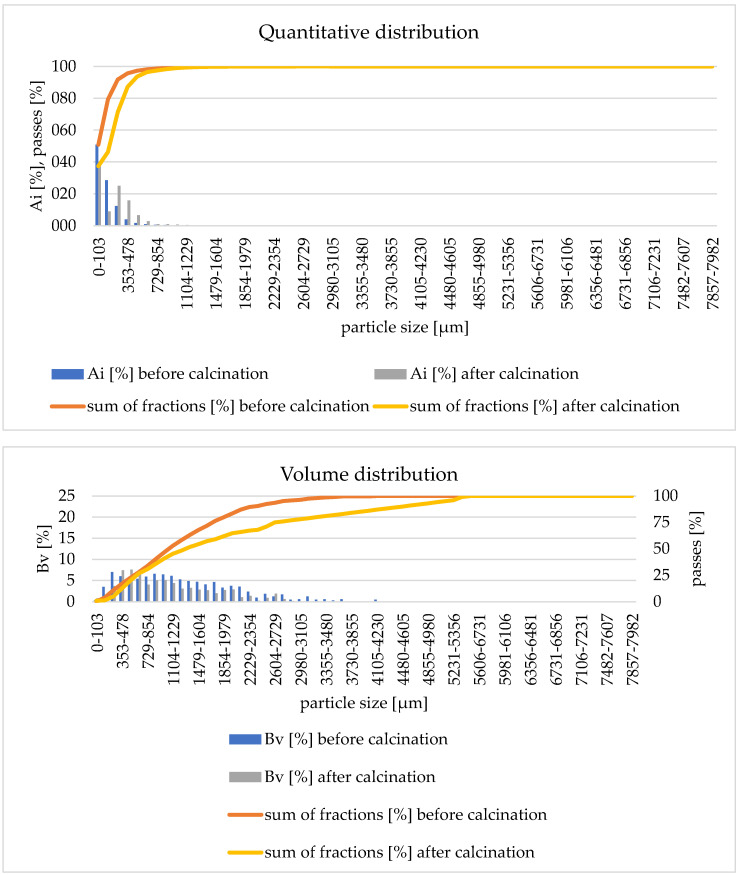
Quantitative and volumetric distribution before and after the calcination process—experiment 4.

The final stage of research verifying the calcination process was the comparison of the particle distribution of the raw material before and after the calcination process. An example of the quantitative and volumetric distribution of granules before and after the calcination process is shown in Figure 10.

**Figure 11:**
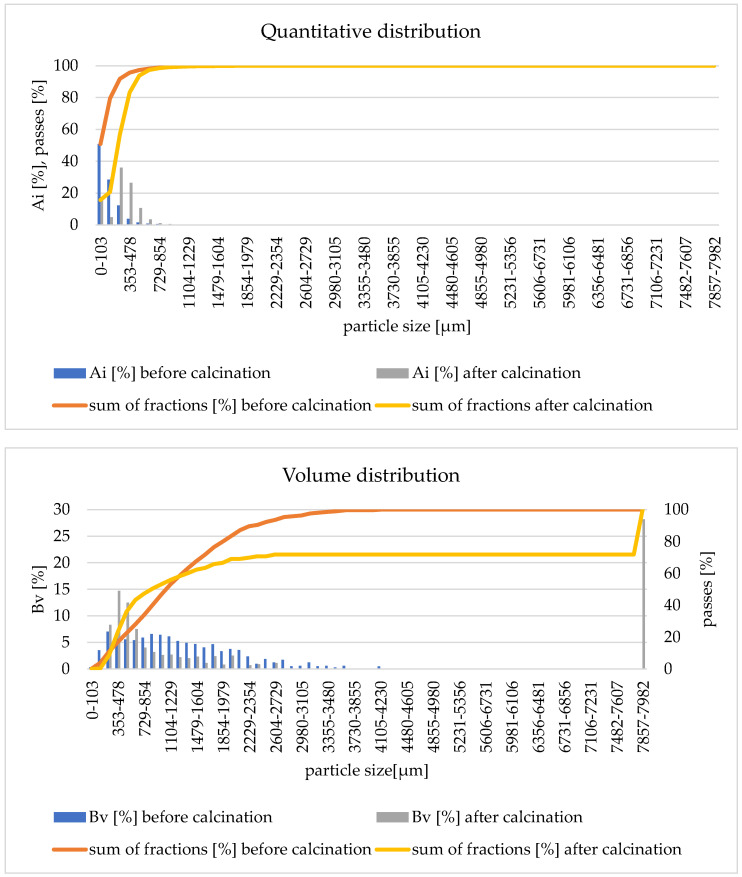
Quantitative and volumetric distribution before and after the calcination process—experiment 11.

The quantitative distribution shows a loss of the finest fraction of particles after the calcination process (in the diameter range <103 µm by approximately 14%). The reason for this situation is the transport of the smallest particles of bulk material together with reaction gases from the reactor. The volumetric distribution shows a shift of the cumulative curve after calcination towards smaller particles. This indicates an increase in the share of particles in the range of 478–729 µm. This means that the granulate particles were fragmented during the fluidized bed calcination process. In the case of the remaining experiments (studies 3, 5–10 and 12), the results of the particle distributions are similar. The formation of fine particles is observed each time. The exception is experiment no. 11 with anthracite 2 with a particle size of 1–1.6 mm (Figure 11) where the formation of sinters in the deposit is observed. The sinters produced are clearly visible in the volume distribution in the particle range of 7857–7982 µm.

**Figure 12:**
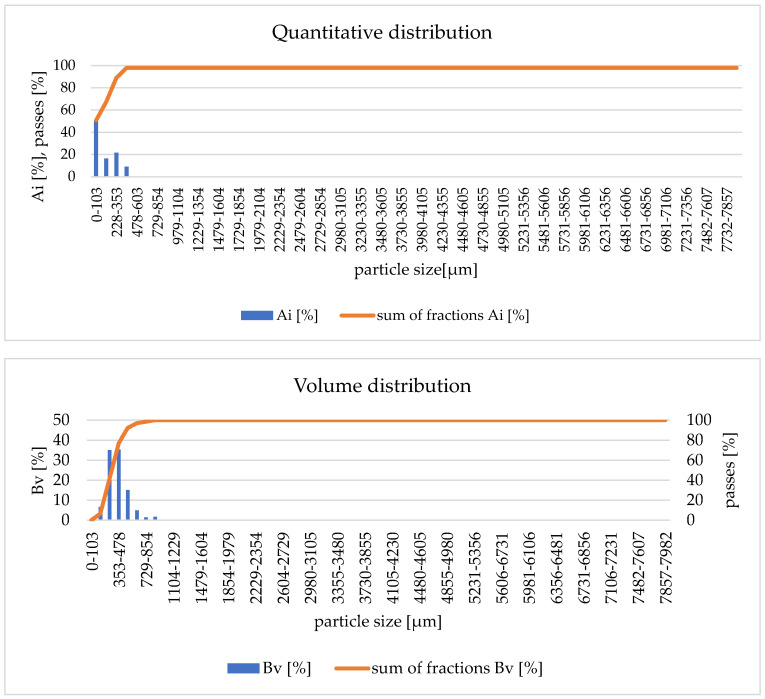
Quantitative and volumetric distribution—subfine particles from the separator.

Due to the observation of small fractions of calcined material being blown out of the main reactor chamber during the calcination tests, it was decided to collect and analyse the blown-out particles. A solid particle separator was installed on the chimney duct used to remove waste gases from both reactor chambers, as indicated in Figure 5. This allowed for the collection of exhausted particles after the calcination experiments of clay materials retained in the separator. The collected particles were analysed for particle distribution, and the results are presented in Figure 12. AWK analysis showed that the particles were in the range of <1354 µm, with nearly 51% of the particles being the <103 µm fraction.

Subsequently, the material was subjected to the determination of the loss on ignition and the degree of calcination. It was determined that the loss on ignition was 0.09% and the degree of calcination was 99.35%. The obtained result of the degree of calcination indicated that despite a small fraction of material being blown out of the reactor chamber, these particles were calcined to almost 100%. This allowed us to conclude that the use of the appropriately selected separators may, in the future, reduce the losses of the smallest fraction of particles during the fluidized bed calcination process. However, the separated material may be used as a fine calcination product.

## 4. Discussion

The calcination of clay raw materials is, and will be in the future, an important process used to produce a number of raw materials beneficial in, among others, the refractory, abrasive, construction and other industries. The use of calcined clay materials is becoming common in construction, where they are used as a pozzolanic component in cement or as a cementing material in concrete to improve its properties.

The method of calcining clay raw materials in a fluidized bed offers potential benefits in terms of energy and fuel savings. This is due to more favourable conditions of heat and mass transfer in fluidized bed conditions, mainly due to the high heat transfer coefficient and intense contact of hot gases with particles of clay material. The use of fluidized bed technology also guarantees an even bed temperature, which allows for the high homogeneity of the obtained calcinate. Additionally, a huge advantage of fluidized bed calcination is the several-fold reduction in process time compared to current methods. Under optimal conditions (fluidized bed temperature 850 °C and material humidity up to 5% [36]), the fluidized bed calcination process can be limited to several minutes.

## 5. Conclusions

As a result of the experimental research carried out as part of this work, the following conclusions were formulated:

(1) The granulation process of clay material facilitates in obtaining material with the desired quantitative and qualitative parameters. Thanks to this, it is possible to obtain a final product with various properties required by their recipients.
(2) Coal fuels can be successfully used in the fluidized bed calcination of clay materials.
(3) When using solid fuels for the fluidized bed calcination process, the particle distribution of the fuels is crucial. The use of coal particles <0.75 mm in the tested fluidized bed calcination process resulted in their blowing out of the reactor chamber, increasing the energy deficit of the process and increasing the loss of incomplete combustion. The use of coal particles >1.6 mm in the fluidized calcination process resulted in insufficient time for their complete combustion. As a result, there was a carbon residue in the final product, which may have disqualified the product.
(4) When anthracite is used as the basic fuel in the fluidized bed thermal calcination process, the control of the bed temperature is a very important factor. At a temperature below the fuel ignition temperature [39], tar may be released on the anthracite surface. As a consequence, this leads to the fusion of clay material particles with fuel particles and the formation of agglomerates or sintered particles, which, in the event of their rapid accumulation, may lead to a deterioration of the fluidization process and even to the defluidization of the bed.
(5) Research on the calcination process using hard coal type 31.2 showed that cheaper and more easily available energetic hard coal can be used. When hard coal was used, neither the release of tar nor the formation of agglomerates was observed.
(6) The particle analysis showed that the clay granulate is fragmented during the fluidized bed calcination process. The first reason is the process of the abrasion of its external surface as a result of the mutual interaction of the particles of clay material granules, and the second is the mechanical impact and the rapid occurrence of dehydration and dehydroxylation processes, which consequently leads to the disintegration of granulate particles.
(7) The analysis showed that despite the smallest fraction of clay granules being blown out of the calcination chamber (particles <1354 µm), these particles are also calcined. The degree of their calcination is very high and amounts to >99%. In the future, the use of the appropriately selected separators may not only reduce the losses of the smallest fraction of particles during the fluidized bed calcination process but may also be used to segregate material particles according to particle size.
(8) The balance calculations presented in the work indicate that almost 50% of the total heat demand in the calcination process is used to heat the air necessary for the fluidization of the bed—Figure 6. The use of lower fluidization velocities for a range of particles smaller than that assumed in the study will result in a significant reduction in energy expenditure on the calcination process.
(9) Due to the high temperature and volume of reaction gases leaving the calcination reactor, it is advisable to use heat exchangers to reduce the moisture of the raw clay.
(10) The positive results of tests on the fluidized bed calcination process using fuels such as anthracite and thermal hard coal indicate purposeful research into the possibility of using other solid fuels in the fluidized bed calcination process, i.e., biomass fuels and other alternative fuels.

## Figures and Tables

**Table 1:** Chemical composition of clay material [43].

Compound	Content, %
SiO_2_	58.50%
Al_2_O_3_	32.70%
Fe_2_O_3_	2.51%
TiO_2_	2.01%
CaO	0.33%
MgO	0.61%
K_2_O	2.99%
Na_2_O	0.11%
other	0.24%
together	100%

**Table 2:** Technical analysis results.

Parameter	Moisture [%]	Volatile Matter Content [%]	Clay Content [%]
value	4.32	1.01	94.67

**Table 3:** Results of calculations of fluidization parameters of clay material.

T [°C]	27	850
Ar [−]	67,645.65	3097.19
Remf [−]	31.96	2.23
Umf [m/s]	0.41	0.25
U_t_ [m/s]	7.35	0.63
U_tr_ [m/s]	5.39	0.81
U_mb_ [m/s]	0.41	0.25

**Table 4:** Results of technical analysis of fuels.

Technical Analysis	Anthracite 1	Hard Coal Type 31.2 (Flaming)	Anthracite 2
Moisture content [%]	2.97	8.12	4.8
Volatile content [%]	9.43	30.30	10.07
Fixed carbon [%]	77.38	47.20	82.68
Ash content [%]	10.22	14.38	2.45
Heat of combustion [MJ/kg]	29.28	26.10	31.54
Calorific value [MJ/kg]	28.27	25.00	31.12

**Table 5:** Results of elemental analysis of fuels.

Fuel	Nitrogen, N [%]	Carbon, C [%]	Hydrogen, H [%]	Sulphur, S [%]
Anthracite 1	0.86	62.45	4.31	0.85
Hard coal type 31.2 (flaming)	0.87	55.81	4.45	1.44
Anthracite 2	0.44	76.45	1.40	0.33

**Table 6:** Results of determining the loss on ignition and the degree of calcination of materials after the calcination process while feeding the station with coal fuels and electricity.

No.	Type of Fuel/Type of Coal	Fluidization Velocity [m/s]	Turning Off the Air Heater	Loss on Ignition [%]	Degree of Calcination [%]
1	Electric power supply	0.26	-	0.22	97.92
2		0.26	-	0.01	99.91
3	Anthracite 1	0.28	NO	not marked	
4		0.28	NO	0.24	97.73
5		0.28	YES	0.45	95.74
6		0.28	NO	0.51	95.18
7		0.28	YES	0.66	93.76
8	Hard coal type 31.2	0.29	NO	0.38	96.41
9		0.28	YES	0.01	99.91
10		0.28	NO	0.06	99.43
11	Anthracite 2	0.29	NO	6.73	36.34
12		0.28	NO	0.66	93.76

## Data Availability

Data are contained within the article.

## References

[1] Ratajczak T., Hycnar E., Bożęcki P. (2016). Kryterium mineralogiczne jako element oceny przydatności niektórych polskich surowców ilastych do budowy przesłon hydroizolacyjnych. Górnictwo Odkryw. Inst. Górnictwa Odkryw. Poltegor-Inst..

[2] Stoch L. (1974). Minerały Ilaste.

[3] Ito A., Wagai R. (2017). Global distribution of clay-size minerals on land surface for biogeochemical and climatological studies. Sci. Data.

[4] Liu X.X., Liu X.W., Hu Y.H. (2015). Investigation of the thermal behaviour and decomposition kinetics of kaolinite. Clay Miner..

[5] Hanein T., Thienel K.C., Zunino F., Marsh A.T., Maier M., Wang B., Canut M., Juenger M.C., Ben Haha M., Avet F. (2022). Clay calcination technology: State-of-the-art review by the RILEM TC 282-CCL. Mater. Struct..

[6] Cao Y., Wang Y., Zhang Z., Ma Y., Wang H. (2021). Recent progress of utilization of activated kaolinitic clay in cementitious construction materials. Compos. Part B Eng..

[7] Nadeem A., Memon S.A., Lo T.Y. (2013). Mechanical performance, durability, qualitative and quantitative analysis of microstructure of fly ash and metakaolin mortar at elevated temperatures. Constr. Build. Mater..

[8] Madandoust R., Mousavi S.Y. (2012). Fresh and hardened properties of self-compacting concrete containing metakaolin. Constr. Build. Mater..

[9] Fernandez R., Martirena F., Scrivener K.L. (2011). The origin of the pozzolanic activity of calcined clay minerals: A comparison between kaolinite, illite and montmorillonite. Cem. Concr. Res..

[10] Małaszkiewicz D. (2015). Metakaolinit jako pucolanowy dodatek do betonu—Przegląd stanu wiedzy. Bud. I Inżynieria Sr..

[11] Ilić B.R., Mitrović A.A., Miličić L.R. (2010). Thermal treatment of kaolin clay to obtain metakaolin. Hem. Ind..

[12] Cassagnabère F., Escadeillas G., Mouret M. (2009). Study of the reactivity of cement/metakaolin binders at early age for specific use in steam cured precast concrete. Constr. Build. Mater..

[13] Neville A.M. (2012). Właściwości Betonu.

[14] De Belie N., Soutsos M., Gruyaert E. (2018). Properties of Fresh and Hardened Concrete Containing Supplementary Cementitious Materials.

[15] Sabir B.B., Wild S., Bai J. (2001). Metakaolin and calcined clays as pozzolans for concrete: A review. Cem. Concr. Compos..

[16] Shah V., Parashar A., Mishra G., Medepalli S., Krishnan S., Bishnoi S. (2020). Influence of cement replacement by limestone calcined clay pozzolan on the engineering properties of mortar and concrete. Adv. Cem. Res..

[17] Ferreiro S., Canut M.M.C., Lund J., Herfort D. (2019). Influence of fineness of raw clay and calcination temperature on the performance of calcined clay-limestone blended cements. Appl. Clay Sci..

[18] Avet F., Scrivener K. (2018). Investigation of the calcined kaolinite content on the hydration of Limestone Calcined Clay Cement (LC3). Cem. Concr. Res..

[19] Zunino F., Martirena Hernandez F., Scrivener K. (2021). Limestone calcined clay cements (LC3). ACI Mater. J..

[20] Khalifa A.Z., Cizer Ö., Pontikes Y., Heath A., Patureau P., Bernal S.A., Marsh A.T. (2020). Advances in alkali-activation of clay minerals. Cem. Concr. Res..

[21] Pouhet R., Cyr M. (2016). Formulation and performance of flash metakaolin geopolymer concretes. Constr. Build. Mater..

[22] Boonjaeng S., Chindaprasirt P., Pimraksa K. (2014). Lime-calcined clay materials with alkaline activation: Phase development and reaction transition zone. Appl. Clay Sci..

[23] (2020). Ruan S et al Solidification of waste excavation clay using reactive magnesia, quicklime, sodium carbonate and early-age oven curing. Constr. Build. Mater..

[24] Piech J. (2001). Piece ceramiczne i Szklarskie.

[25] Teklay A., Yin C., Rosendahl L., Køhler L.L. (2015). Experimental and modeling study of flash calcination of kaolinite rich clay particles in a gas suspension calciner. Appl. Clay Sci..

[26] Gonzalez R.S., Flamant G. (2014). Technical and economic feasibility analysis of using concentrated solar thermal technology in the cement production process: Hybrid approach—A case study. J. Sol. Energy Eng..

[27] Abanades S., André L. (2018). Design and demonstration of a high temperature solar-heated rotary tube reactor for continuous particles calcination. Appl. Energy.

[28] Makul N., Rattanadecho P., Agrawal D.K. (2014). Applications of microwave energy in cement and concrete—A review. Renew. Sustain. Energy Rev..

[29] Reinosa J.J., García-Baños B., Catalá-Civera J.M., Fernández J.F. (2019). A step ahead on efficient microwave heating for kaolinite. Appl. Clay Sci..

[30] Burman T., Engvall J. (2019). Evaluation of Usage of Plasma Torches in Cement Production. Master’s Thesis.

[31] Kurdowski W. (2004). Kilka uwag o piecach wapienniczych. Cem. Wapno Beton.

[32] Khadilkar A., Rozelle P.L., Pisupati S.V. (2014). Models of agglomerate growth in fluidized bed reactors: Critical review, status and applications. Powder Technol..

[33] Scala F., Salatino P. (2003). Dolomite attrition during fluidized-bed calcination and sulfation. Combust. Sci. Technol..

[34] Zhang W. (2009). A Review of Techniques for the Process Intensification of Fluidized Bed Reactors. Chin. J. Chem. Eng..

[35] Shuai Y., Yuexin H., Yanjun L., Peng G., Jianwen Y. (2018). Effect of calcination temperature on activation behaviors of coal-series kaolin by fluidized bed calcination. Physicochem. Probl. Miner. Process..

[36] Kaczyńska K., Kaczyński K., Pełka P. (2021). Calcination of Clay Raw Materials in a Fluidized Bed. Materials.

[37] Gupta J.S., Rao A.V.R. (1978). Production of clay pozzolana by fluidized bed technique. Trans. Indian Ceram. Soc..

[38] Emmanuel A.C., Haldar P., Maity S., Bishnoi S. (2016). Second pilot production of limestone calcined clay cement in India: The experience. Indian Concr. J..

[39] Prins W., Siemons R., Van Swaaij W.P.M., Radovanovic M. (1989). Devolatilization and ignition of coal particles in a two-dimensional fluidized bed. Combust. Flame.

[40] Wójcicki S. (1969). Spalanie.

[41] Rashad A.M. (2013). Metakaolin as cementitious material: History, scours, production and composition—A comprehensive overview. Constr. Build. Mater..

[42] Wyrwicki R. (1998). Analiza Deriwatograficzna Skał Ilastych.

[43] (2017). Internal Report CEWAP sp. z o.o.

[44] (1980). Polish Standard: Solid Fuels. Determination of Moisture Content.

[45] (1981). Polish Standard: Solid Fuels. Determination of Volatile Matter Content.

[46] (1980). Polish Standard: Solid Fuels. Determination of Ash by Weight.

[47] (2013). Tests for Mechanical and Physical Properties of Aggregates—Part 3: Determination of Loose Bulk Density and Voids.

[48] Basu P., Fraser S.A. (1991). Circulating Fluidized Bed Boilers, Design and Operations.

[49] Geldart D. (1973). Types of Gas Fluidization. Powder Technol..

[50] Grammelis P., Margaritis N., Karampinis E., Oakey J. (2016). 2—Solid fuel types for energy generation: Coal and fossil carbon-derivative solid fuels. Fuel Flexible Energy Generation.

